# Commentary: Perspectives from Argentina: Children and agricultural health and safety

**DOI:** 10.3389/fpubh.2022.1015233

**Published:** 2022-12-06

**Authors:** Marcos Grigioni

**Affiliations:** Independent Researcher, Rosario, Argentina

**Keywords:** rural life, adolescents, children's incidents, agricultural health and safety, Argentina current situation

## Current situation

Argentina, located in South America, with a population of 47,327,407 (1), is a farming and ranching country. Food production has an important and strategic place within the current economic situation given that it accounts for 15% of its GDP and 48% of its total exports (2).

Argentina lacks a unified consensus about the age limits for defining children or adolescents in the data collected. Although, information obtained from different sources helps clarify descriptions of children and adolescents living in rural areas. The national agricultural census provides a lower-bound estimate of children who might be exposed to agricultural risks. In 2018, there were 48,984 females and 51,610 males, under age 14 years living on farms (3). Meanwhile the Survey of Activities of Children and Adolescents provides a higher-bound estimate of children who might be exposed to agricultural risks. In 2017, 1,043,949 children (between 5 and 15 years-of-age) lived in rural areas, of which ~207,000 worked performing intensive domestic activities, agricultural tasks to support production for market or household consumption. Of the 194,236 adolescents aged 16 and 17 who live in rural areas, 44% carry out some work related to agricultural production (4).

Regarding rates of injuries to children, surveillance data are currently not available, preventing the drawing of clear conclusions for the purposes of this commentary (5). This is because while the Superintendent of Occupational Risks provides yearly data on work-related accidents for individuals aged 16–24, the data by incident is not disaggregated by age—preventing us from determining injuries impacting adolescents vs. young adults. The Directorate of Statistics and Health Information publishes reports on the causes of death of individuals according to age, sex, and province, but it does not determine if the death occurred in rural areas or if the person was a rural resident, farmworker, or farmer.

Although there are no official statistics on injuries in rural areas, journalistic sources provide insights. The majority of rural injuries in children and adolescents occurred during recreational activities, work, when accompanying working family members, or as a consequence of living in the countryside. Data collected by this author show that in 2013, 33 children under age 16 died, and 44 died in 2014 from injuries and diseases related to some agricultural/rural activity (6). In the last 5 years, another 229 cases of fatalities in children under age 17 were reported in the media. This information is surely incomplete, and likely under reported, because not all situations are published or reported.

The analysis of the cases published in the media shows the characteristics of rural injuries involving children are closely related to the region of the country where they occur. In the so-called “core zone,” motor vehicle-crashes on rural roads or routes are most prevalent, involving children accompanying their parents for work. Changes in agricultural production including through the increasing use of high technology (i.e., direct sowing) and the consolidation in agricultural production means that families have been able to move away from their rural residences to live in nearby towns or cities. Therefore, farmers travel several times a week to the field to complete tasks and then return to town, increasing the weekly kilometers traveled and increasing the risk of a road accident. In other regions of the country, such as the Northwest, Northeast, Cuyo, and Patagonia, where the production requires permanent labor (livestock, fruit trees, orchards, yerbatales, etc.), the farmers and workers with their families are more permanent and reside on the farm. There the injuries to youth are related to things such as animals (mainly horses), drowning, and machinery (frequently the tractor).

## Current activities

Laws and regulations protect children from child labor. In Argentina, child labor is defined as “any productive or service activity in which girls or boys under 16 years-of-age participate, regardless of their employment status (whether they receive payment for their work or not, be it family or for third parties), that hinders their schooling and/or that due to their environment or conditions implies current or future damage to their health and psychophysical development” (7). There are however, exceptions to this law, boys and girls between the ages of 14 and 15 whose parents operate a family business can work given the provisions of protective measures, such as a 3-h workday. Adolescent work (16 and 17 years old) is allowed but they are offered protections including: working no more than 6 h a day or 36 h a week; no work before 6 a.m. or after 8 p.m.; no work that is considered dangerous, arduous or unhealthy (8).

A few initiatives protect children from rural injuries. The RENATRE (National Registry of Rural Workers and Unloaders) has opened houses to accommodate minors while their parents work in the harvest in certain parts of the country (Grow Program) and thus distance them from the risks present in the workplace. Also since 2021, the government, together with unions and companies, promote the creation of Rural Socio-Educational Centers (CSER). These centers house the children of seasonal and migrant rural workers under 16 years during the harvest months. For example, in the Mendoza province, there are 48 of these centers, accommodating more than 3,000 children and adolescents under age 16. Children from other provinces such as Misiones, Salta, Tucumán, Jujuy, have benefited from this endeavor (9).

## Current challenges

There are many challenges to protect the children in the field. Taking a closer look at the existing government actions to prevent rural injuries in children and adolescents, the majority are focused and directed only at children of temporary or migrant rural workers and adolescent workers covered by the work insurance system, leaving out of these initiatives other children and adolescents who live in or visit rural areas, for example children of farmers, children traveling for rural tourism, etc. Furthermore, the preventative activities financed and organized by agricultural machinery companies, agricultural foundations, and NGOs can largely be characterized as isolated, sporadic, and temporary initiatives without continuity. Often the objective is to improve the image of the organizer rather than a social service to prevent childhood injuries. Groups of rural women, from their agricultural cooperatives, have supported and developed education and training programs for producers, workers, and rural families to prevent injuries involving children, covering a very large gap that exists linked to the dissemination of preventive information.

Several important actors are also not currently addressing rural injury prevention which represent missed opportunities. This includes public organizations related to rural, the curriculum in rural and agritechnical schools, most government manuals and technical publications of companies or private institutions for the development of agricultural activities (such as agritourism and rural exhibitions). This also includes the lack of training or research in agricultural health and safety in medical schools, except for issues related to poisoning with agrochemicals. Positively, the SAP (Society of Argentine Pediatrics), does list the risks and ways to prevent injuries in rural areas in its Accident Prevention Manual (10).

Last, shortcomings in the prevention of rural injuries in children are observed daily in Argentina. For example, often in the media, images of children in risky situations in the field are shown as something charming or normal. In turn, the sensationalism of publishing news about a fatal case in the field is accompanied by varied details (episodic stories), but without preventive recommendations for readers (absence of thematic stories). This is different than the reported news about roadway crashes, where information about risks is frequently included and detailed, and the causes are analyzed. It seems media ignore or minimize their power to help reduce injuries in agriculture. However, this could also be due to lack of knowledge of the subject.

## Future direction

A range of changes is needed to improve the safety of rural Argentinian children. It is first necessary to improve the official registries to understand the prevalence and the types of injuries. This information is important for the development and refinement of agricultural health and safety interventions. Developing government programs on agricultural health and safety and injuries prevention is also urgently needed in Argentina. Having teachers at the primary (rural schools) and secondary level (agritechnical schools) educate their students in injury prevention could lead thousands of children to a safer childhood and adolescence, transferring this knowledge to their children in the future. This could help address the challenges around the risky habits and customs, so ingrained in Argentine country life. The creation of university study programs in agricultural health and safety for the qualification and training of health professionals in said area is relevant. Besides educational programs, the provision of childcare should be a fundamental pillar. All the proposed improvements require joint interdisciplinary and cooperative work between government agencies, educational institutions at all levels (schools, colleges, and universities), media, unions of rural workers and farmer's associations to be able to face and improve the dire statistics of childhood injuries that impact the Argentine countryside year after year. The private sector (e.g., agricultural machinery companies), through its corporate social responsibility, can complement these initiatives by contributing funds or material and human resources. Last, the media can contribute to increased injury prevention measures in different ways. One of them would be use images showing no risky situations for children. Another would be the correct use of language (for example, “incidents” instead of “accidents”), along with the incorporation of recommendations for the prevention of these incidents. The basis of all this contribution should begin with the training of journalists on this problem.

## Author contributions

The author confirms being the sole contributor of this work and has approved it for publication.

## Funding

The Marshfield Clinic Research Institute provided financial support by paying for the publication fees (Frontiers grant: code discount: DSC-08033864004PRD).

## Conflict of interest

The author declares that the research was conducted in the absence of any commercial or financial relationships that could be construed as a potential conflict of interest.

## Publisher's note

All claims expressed in this article are solely those of the authors and do not necessarily represent those of their affiliated organizations, or those of the publisher, the editors and the reviewers. Any product that may be evaluated in this article, or claim that may be made by its manufacturer, is not guaranteed or endorsed by the publisher.
